# Health and Economic Value of Eliminating Socioeconomic Disparities in US Youth Physical Activity

**DOI:** 10.1001/jamahealthforum.2024.0088

**Published:** 2024-03-15

**Authors:** Tiffany M. Powell-Wiley, Marie F. Martinez, Jessie Heneghan, Colleen Weatherwax, Foster Osei Baah, Kavya Velmurugan, Kevin L. Chin, Colby Ayers, Manuel A. Cintron, Lola R. Ortiz-Whittingham, Dana Sandler, Sonal Sharda, Meredith Whitley, Sarah M. Bartsch, Kelly J. O’Shea, Alexandra Tsintsifas, Alexis Dibbs, Sheryl A. Scannell, Bruce Y. Lee

**Affiliations:** 1 Social Determinants of Obesity and Cardiovascular Risk Laboratory, Cardiovascular Branch, Division of Intramural Research, National Heart, Lung, and Blood Institute, National Institutes of Health, Bethesda, Maryland; 2 Intramural Research Program, National Institute on Minority Health and Health Disparities, National Institutes of Health, Bethesda, Maryland; 3 Public Health Informatics, Computational, and Operations Research (PHICOR), CUNY Graduate School of Public Health and Health Policy, New York, New York; 4 Center for Advanced Technology and Communication in Health (CATCH), CUNY Graduate School of Public Health and Health Policy, New York, New York; 5 Artificial Intelligence, Modeling, and Informatics, for Nutrition Guidance and Systems (AIMINGS) Center, New York, New York; 6 Nell Hodgson Woodruff School of Nursing, Emory University, Atlanta, Georgia; 7 Ruth S. Ammon College of Education and Health Sciences, Adelphi University, Garden City, New York; 8 Maties Sport, Centre for Sport Leadership, Stellenbosch University, Stellenbosch, South Africa

## Abstract

**Importance:**

There are considerable socioeconomic status (SES) disparities in youth physical activity (PA) levels. For example, studies show that lower-SES youth are less active, have lower participation in organized sports and physical education classes, and have more limited access to PA equipment.

**Objective:**

To determine the potential public health and economic effects of eliminating disparities in PA levels among US youth SES groups.

**Design and Setting:**

An agent-based model representing all 6- to 17-year-old children in the US was used to simulate the epidemiological, clinical, and economic effects of disparities in PA levels among different SES groups and the effect of reducing these disparities.

**Main Outcomes and Measures:**

Anthropometric measures (eg, body mass index) and the presence and severity of risk factors associated with weight (stroke, coronary heart disease, type 2 diabetes, or cancer), as well as direct and indirect cost savings.

**Results:**

This model, representing all 50 million US children and adolescents 6 to 17 years old, found that if the US eliminates the disparity in youth PA levels across SES groups, absolute overweight and obesity prevalence would decrease by 0.826% (95% CI, 0.821%-0.832%), resulting in approximately 383 000 (95% CI, 368 000-399 000) fewer cases of overweight and obesity and 101 000 (95% CI, 98 000-105 000) fewer cases of weight-related diseases (stroke and coronary heart disease events, type 2 diabetes, or cancer). This would result in more than $15.60 (95% CI, $15.01-$16.10) billion in cost savings over the youth cohort’s lifetime. There are meaningful benefits even when reducing the disparity by just 25%, which would result in $1.85 (95% CI, $1.70-$2.00) billion in direct medical costs averted and $2.48 (95% CI, $2.04-$2.92) billion in productivity losses averted. For every 1% in disparity reduction, total productivity losses would decrease by about $83.8 million, and total direct medical costs would decrease by about $68.7 million.

**Conclusions and Relevance:**

This study quantified the potential savings from eliminating or reducing PA disparities, which can help policymakers, health care systems, schools, funders, sports organizations, and other businesses better prioritize investments toward addressing these disparities.

## Key Points

**Question:**

What are the potential public health and economic effects of eliminating disparities in physical activity (PA) levels among US youth socioeconomic status (SES) groups?

**Findings:**

This study used an agent-based model to test the estimated effect of eliminating the SES disparity in youth PA levels and found that it would avert 383 000 overweight and obesity cases and 101 000 weight-related disease cases (stroke/coronary heart disease events, type 2 diabetes, or cancer), resulting in more than $15.6 billion in cost savings over the youth cohort’s lifetime.

**Meaning:**

This study quantified the potential savings from eliminating or reducing PA disparities, which can help policymakers better prioritize investments toward addressing these disparities.

## Teaser

This study aims to determine the potential public health and economic effects of eliminating disparities in physical activity levels among US youth socioeconomic status groups.

## Introduction

There are considerable socioeconomic status (SES) disparities in youth physical activity (PA) levels in the US. For example, in low-SES schools, only 24.6% of eighth graders play sports.^1^ Another study reports that school sport participation correlates negatively with SES and was lower among low-SES youth.^2^ This study also found that lower-SES students were less likely to have school physical education requirements and that lower SES was associated with lower participation in physical education.^2^ Research on Wisconsin students found that those in lower-income schools were less active than those in higher-income schools (68.2 vs 73.3 minutes) and scored lower on fitness tests.^3^ A study of children in Seattle, Washington, and San Diego, California, found that lower-income youth had lower access to portable play equipment (eg, bicycles, jump ropes) compared with higher-income children.^4^ Such disparities in PA during childhood/adolescence can contribute to health disparities, with lower-income youth being at higher risk for negative health outcomes in adulthood (eg, coronary heart disease [CHD],^5^ stroke,^6,7^ diabetes,^8^ and cancer^9,10,11^).^12,13^ There is one remaining question: what would happen if we eliminated these PA disparities and achieved parity in PA levels across SES groups? Understanding and quantifying the potential health and economic benefits of this could help policymakers better allocate their limited resources toward interventions aimed at eliminating PA disparities, which could subsequently reduce long-term health care expenditures and improve population-level health. Therefore, this study uses an agent-based model (ABM) to demonstrate and quantify the estimated health and economic effect of achieving parity in PA across SES groups for US children and adolescents aged 6 to 17 years.

## Methods

### ABM Overview

**Figure 1.**
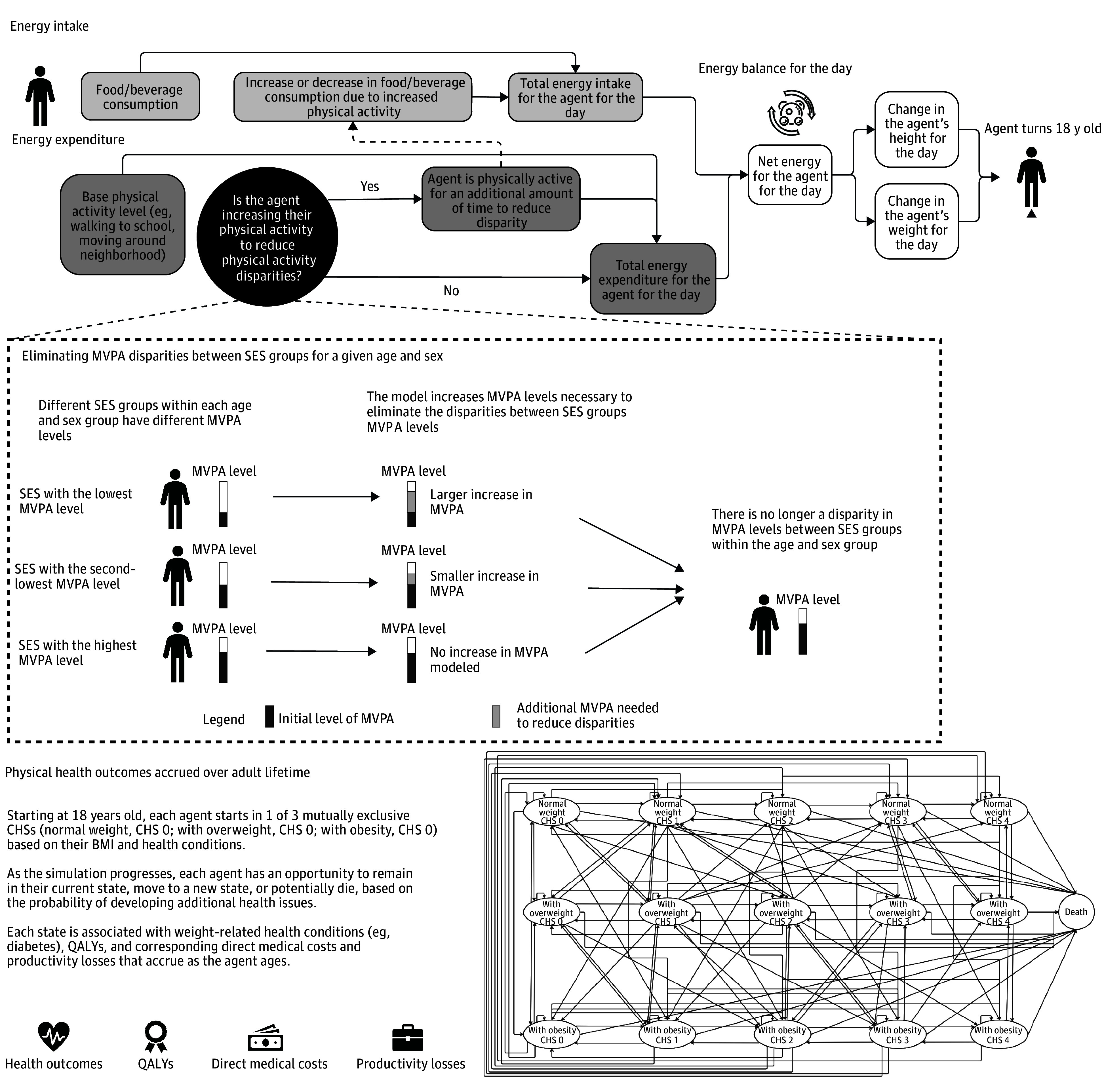
Youth Physical Activity Disparity Model BMI indicates body mass index (calculated as weight in kilograms divided by height in meters squared); CHS, chronic health state; MVPA, moderate to vigorous physical activity; QALYs, quality-adjusted life-years; SES, socioeconomic status.

Using the previously described Virtual Population for Obesity Prevention ABM,^14,15,16,17,18^ we represented all 50 million US children and adolescents 6 to 17 years old (starting in 2023), their growth over time, daily levels of PA, and physical health outcomes. The model represents each child as a computational agent and simulates each day of their childhood/adolescent years until age 18 years, then each year for the rest of their lives. Similar to a real child, each agent has a set of sociodemographic characteristics including age, sex, and SES (represented as 4 categories, defined based on household income percentage compared with the federal poverty level [FPL]), as well as clinical characteristics (fat-free mass, fat mass^19^). Figure 1 outlines how each agent proceeds through the model. Each day, each agent grows in height based on nationally representative growth charts^20^ and consumes calories to maintain a constant body mass index (BMI; calculated as weight in kilograms divided by height in meters squared) percentile if their PA level was unchanged. This study was exempt from institutional review board approval, as it does not involve active data collection nor interaction with human participants. This study adheres to the Consolidated Health Economic Evaluation Reporting Standards (CHEERS) 2022 checklist for decision analytical models.

### Metabolic Model Embedded in Each Agent

Each agent has an embedded metabolic model specific to their age, sex, and weight. The metabolic model tracks their caloric consumption and energy expenditure and translates caloric surplus or deficit into weight gain or weight loss each day.^21,22^

### Representing Each Agent’s PA

As Figure 1 shows, at the simulation start, each agent has a range of daily PA levels based on their age, sex, and SES, which translates to caloric expenditures. Each week, each agent draws a certain number of days per week that they get 60 minutes of PA,^23^ which varies by age, sex, and SES. We assumed that youth get 30 minutes of PA on the remaining days in the week (varied in sensitivity analyses). In general, youth with a higher SES get more PA and are active for 60 minutes on more days per week compared with youth in the lower-SES groups (Table 1). Thus, changes in PA for any agent result in weight gain or loss, each day up until age 18 years, specific to that person’s change in PA and their BMI.

**Table 1.** Youth Physical Activity and Anthropometric Measures by Age, Sex, and Socioeconomic Status^a^

Federal poverty level	Mean (SE)					
	Males			Females		
	6-10 y Old	11-13 y Old	14-17 y Old	6-10 y Old	11-13 y Old	14-17 y Old
**No. in population (n = 49 878 670)**						
<100%	1 968 958	1 286 059	1 572 564	1 570 444	1 071 792	1 690 081
100%-199%	2 152 037	1 509 539	1 874 065	2 084 059	1 414 506	1 715 986
200%-399%	3 019 002	1 788 511	2 532 555	2 889 740	1 892 410	2 439 551
≥400%	3 185 931	1 837 003	2 810 412	3 315 163	1 734 083	2 524 229
**Average No. of days per week physically active for ≥60 min**						
<100%	3.97 (0.14)	3.26 (0.18)	3.20 (0.14)	3.68 (0.14)	2.80 (0.19)	2.64 (0.15)
100%-199%	3.90 (0.13)	3.44 (0.13)	3.01 (0.12)	3.83 (0.11)	2.70 (0.13)	2.44 (0.11)
200%-399%	4.16 (0.09)	3.64 (0.10)	3.42 (0.08)	3.95 (0.08)	3.11 (0.10)	2.75 (0.08)
≥400%	4.39 (0.06)	3.91 (0.09)	3.70 (0.06)	4.11 (0.06)	3.38 (0.07)	3.01 (0.06)
**Overweight prevalence, %**						
<100%	18.18 (3.48)	18.76 (3.23)	12.36 (1.46)	21.40 (6.06)	24.19 (3.56)	15.03 (2.44)
100%-199%	19.97 (3.34)	21.97 (3.15)	19.18 (2.50)	25.48 (6.90)	19.95 (2.91)	15.49 (2.43)
200%-399%	17.84 (2.39)	19.64 (2.01)	13.58 (1.10)	17.05 (3.03)	18.22 (2.27)	14.78 (1.29)
≥400%	18.23 (2.35)	15.26 (1.19)	13.38 (1.55)	14.91 (2.53)	13.79 (1.24)	12.08 (1.06)
**Obesity prevalence, %**						
<100%	34.29 (5.08)	27.71 (3.22)	22.12 (2.37)	28.30 (5.56)	24.13 (2.99)	20.59 (2.49)
100%-199%	25.17 (4.02)	24.38 (2.69)	25.14 (2.49)	17.52 (3.29)	17.33 (2.55)	21.23 (2.60)
200%-399%	29.14 (4.03)	18.79 (1.82)	20.26 (1.79)	18.41 (2.98)	13.17 (1.62)	12.91 (1.61)
≥400%	16.05 (2.22)	10.26 (0.97)	10.77 (0.83)	9.24 (2.39)	6.64 (0.73)	6.31 (0.61)

^a^ Data are from the 2021 National Survey of Children’s Health.^23^

### Representing the Physical Health Outcomes for Each Agent

Starting at age 18 years, agents enter a Markov model (Figure 1), described in previous publications,^15,16,17^ which consists of 15 mutually exclusive health states accounting for both anthropometric measures (eg, BMI) and the presence and severity of risk factors associated with weight. Agents start at a metabolically healthy state and assume 1 of 3 states based on their BMI category (normal weight, overweight, or with obesity) at the end of childhood.

Each simulated year, the agent has probabilities of staying in the same health state or moving to a new health state based on state-, age-, and sex-specific probabilities. While in each health state, the agent has probabilities of developing weight-related health outcomes such as stroke, CHD, type 2 diabetes, and cancers, and accrues health state–, health outcome–, and age-specific medical costs, lost productivity, and quality-adjusted life-years (eTables 1-3 in Supplement 1). Individuals accrue costs throughout their entire lifespan; thus, those who live longer and experience more health outcomes will accrue more costs than those dying younger. The probabilities for these health outcomes differ for each BMI category. Thus, weight loss in childhood/adolescence causing a change in BMI categories will ultimately affect the individual’s probability of experiencing health outcomes throughout adulthood. However, to remain conservative, if a child/adolescent loses weight but does not change BMI categories, they will not experience a change in these outcome probabilities.

### Economic Outcomes

The third-party payer perspective includes direct medical costs, while the societal perspective includes direct and indirect (ie, productivity losses due to presenteeism) costs. Daily wage attenuated by the individual’s health condition–specific utility weight for the duration of their condition serves as a proxy for productivity losses and is calculated as follows:

Daily wage × (1 − utility weight) × duration of outcome

All individuals accrue productivity losses, regardless of age or employment status, since everyone contributes to society. We report all costs in 2023 US dollars, converting all past and future costs using a 3% annual rate. Similarly, all future quality-adjusted life-years are presented in net present value, discounted with a 3% rate.

### Experimental Scenarios and Sensitivity Analysis

Different experiments simulated the effect of reducing, to varying degrees, the disparities in PA levels between different age, sex, and SES groups. The first set of scenarios brings each age, sex, and SES group to the highest level of PA observed across their sex and age group. For example, for boys 6 to 10 years old, the highest PA level in that group was among those 400% FPL or higher at 4.4 days per week physically active. Therefore, we brought all boys 6 to 10 years old up to an average of 4.4 days per week to eliminate the disparity within that group. We repeated this for the other groups. Next, we simulated the effect of reducing disparities among SES groups within each age and sex group by 75%, 50%, and 25%. The baseline scenario assumed that the influence of additional PA on the weight of individuals was not affected by SES. Studies have shown that those in lower SES groups may face additional barriers to losing weight (eg, living in neighborhoods oversaturated with unhealthy food options).^24,25^ At the same time, evidence exists showing that increasing PA can result in other lifestyle, behavioral, and social changes (eg, healthier eating behaviors) that can facilitate weight loss.^26,27^ Therefore, sensitivity analyses varied the accompanying changes in calorie consumption that may occur with increases in PA, ranging from a 2% decrease in caloric consumption (representing healthier diets) to an increase in caloric consumption equivalent to 50% of calories expended from additional PA (representing compensatory eating or consumption of more caloric dense food/beverages such as soda and ultraprocessed food). Sensitivity analyses also varied the amount of PA that the agents got on the days when they were not getting at least 60 minutes (eg, 0-45 minutes). Since the goal of this study was to determine what would happen if SES disparities in average amounts of PA were eliminated or reduced, we assumed in all experimental scenarios that individuals maintained increases in PA until age 18 years. Analyses were completed using Python, version 3.7 (Python Software Foundation), and NumPy, version 1.19.

## Results

### Estimated Physical Health Effects of Reducing Disparities in PA Levels Among Youth in Different SES Groups Nationwide

Table 2 summarizes the physical health outcomes that result from reducing disparities in PA levels among youth in different SES groups. For example, this generates a decrease in absolute overweight/obesity prevalence by 0.826% (95% CI, 0.821%-0.832%) among youth 6 to 17 years old (a reduction from 35.6%, the cohort’s baseline overweight/obesity prevalence), which results in approximately 383 000 (95% CI, 368 000-399 000) fewer cases of overweight and obesity. Additionally, eliminating the SES disparities within all youth age and sex groups averts 101 000 (95% CI, 98 000-105 000) cases of weight-related diseases over their lifetime, reducing health disparities such as diabetes prevalence by 0.26%, cancer by 0.05%, CHD deaths by 0.49%, and diabetes deaths by 0.75% across the cohort.

**Table 2.** Number and Relative Percentage of Clinical Outcomes Averted When Reducing Physical Activity Disparities Among US Youth^a^

Reduction in disparities	Females			Males			Total
	6-10 y Old	11-13 y Old	14-17 y Old	6-10 y Old	11-13 y Old	14-17 y Old	
**Health effects**							
**Quality-adjusted life-years saved over lifetime**							
25%							
Mean (95% CI)	8000 (2000 to 14 000)	−2000 (−7000 to 3000)	1000 (−5000 to 7000)	43 000 (38 000 to 49 000)	10 000 (6000 to 14 000)	21 000 (16 000 to 25 000)	82 000 (69 000 to 95 000)
% (95% CI)	0.004 (0.001 to 0.006)	−0.002 (−0.007 to 0.003)	0.001 (−0.003 to 0.004)	0.017 (0.015 to 0.020)	0.009 (0.005 to 0.013)	0.010 (0.008 to 0.012)	0.008 (0.006 to 0.009)
50%							
Mean (95% CI)	34 000 (28 000 to 41 000)	20 000 (15 000 to 26 000)	6000 (0 to 13 000)	70 000 (64 000 to 75 000)	36 000 (31 000 to 40 000)	27 000 (22 000 to 32 000)	193 000 (180 000 to 207 000)
% (95% CI)	0.015 (0.012 to 0.018)	0.020 (0.015 to 0.026)	0.003 (0.000 to 0.007)	0.028 (0.026 to 0.030)	0.033 (0.028 to 0.036)	0.013 (0.010 to 0.015)	0.018 (0.017 to 0.019)
75%							
Mean (95% CI)	45 000 (38 000 to 52 000)	43 000 (38 000 to 49 000)	11 000 (5000 to 17 000)	98 000 (92 000 to 104 000)	48 000 (44 000 to 53 000)	40 000 (35 000 to 45 000)	286 000 (272 000 to 299 000)
% (95% CI)	0.020 (0.017 to 0.023)	0.042 (0.037 to 0.048)	0.006 (0.003 to 0.009)	0.040 (0.037 to 0.042)	0.043 (0.040 to 0.048)	0.019 (0.017 to 0.021)	0.026 (0.025 to 0.027)
100%							
Mean (95% CI)	53 000 (47 000 to 59 000)	53 000 (47 000 to 59 000)	20 000 (14 000 to 26 000)	113 000 (108 000 to 119 000)	55 000 (51 000 to 60 000)	63 000 (58 000 to 68 000)	357 000 (344 000 to 371 000)
% (95% CI)	0.023 (0.021 to 0.026)	0.052 (0.046 to 0.058)	0.010 (0.007 to 0.013)	0.046 (0.044 to 0.048)	0.050 (0.046 to 0.054)	0.030 (0.028 to 0.032)	0.033 (0.032 to 0.034)
**Years of life saved over lifetime**							
25%							
Mean (95% CI)	−2000 (−33 000 to 30 000)	2000 (−24 000 to 28 000)	−5000 (−36 000 to 26 000)	24 000 (−8000 to 55 000)	5000 (−23 000 to 33 000)	22 000 (−8000 to 53 000)	47 000 (−26 000 to 120 000)
% (95% CI)	−0.001 (−0.011 to 0.010)	0.001 (−0.008 to 0.010)	−0.001 (−0.007 to 0.005)	0.003 (−0.001 to 0.008)	0.002 (−0.007 to 0.010)	0.004 (−0.001 to 0.009)	0.002 (−0.001 to 0.004)
50%							
Mean (95% CI)	8000 (−24 000 to 40 000)	−7000 (−35 000 to 20 000)	−20 000 (−53 000 to 12 000)	30 000 (−3000 to 62 000)	5000 (−22 000 to 32 000)	6000 (−25 000 to 38 000)	21 000 (−53 000 to 96 000)
% (95% CI)	0.003 (−0.008 to 0.014)	−0.002 (−0.012 to 0.007)	−0.004 (−0.010 to 0.002)	0.004 (0.000 to 0.009)	0.002 (−0.007 to 0.010)	0.001 (−0.004 to 0.006)	0.001 (−0.002 to 0.003)
75%							
Mean (95% CI)	28 000 (−4000 to 60 000)	39 000 (11 000 to 68 000)	19 000 (−13 000 to 50 000)	48 000 (14 000 to 82 000)	20 000 (−8000 to 47 000)	64 000 (34 000 to 95 000)	217 000 (142 000 to 292 000)
% (95% CI)	0.010 (−0.001 to 0.021)	0.013 (0.004 to 0.023)	0.003 (−0.002 to 0.009)	0.007 (0.002 to 0.011)	0.006 (−0.002 to 0.015)	0.011 (0.006 to 0.016)	0.007 (0.005 to 0.009)
100%							
Mean (95% CI)	40 000 (8000 to 73 000)	−11 000 (−38 000 to 16 000)	4000 (−25 000 to 33 000)	61 000 (26 000 to 96 000)	33 000 (7000 to 60 000)	63 000 (32 000 to 95 000)	191 000 (116 000 to 266 000)
% (95% CI)	0.014 (0.003 to 0.025)	−0.004 (−0.013 to 0.006)	0.001 (−0.005 to 0.006)	0.009 (0.004 to 0.013)	0.010 (0.002 to 0.019)	0.010 (0.005 to 0.016)	0.006 (0.004 to 0.009)
**Overweight and obesity cases averted between ages 6 and 17 y**							
25%							
Mean (95% CI)	9000 (5000 to 13 000)	5000 (4000 to 6000)	0 (−1000 to 1000)	32 000 (25 000 to 39 000)	17 000 (15 000 to 19 000)	23 000 (19 000 to 43 000)	86 000 (76 000 to 95 000)
% (95% CI)	0.05 (0.03 to 0.07)	0.25 (0.20 to 0.30)	0.00 (−0.01 to 0.01)	0.13 (0.10 to 0.15)	0.70 (0.62 to 0.78)	0.12 (0.10 to 0.23)	0.11 (0.09 to 0.12)
50%							
Mean (95% CI)	33 000 (28 000 to 37 000)	16 000 (13 000 to 20 000)	3000 (1000 to 5000)	47 000 (39 000 to 56 000)	40 000 (35 000 to 44 000)	38 000 (35 000 to 43 000)	177 000 (165 000 to 190 000)
% (95% CI)	0.17 (0.15 to 0.20)	0.80 (0.65 to 1.00)	0.02 (0.01 to 0.04)	0.19 (0.15 to 0.22)	1.64 (1.44 to 1.81)	0.20 (0.18 to 0.23)	0.14 (0.20 to 0.23)
75%							
Mean (95% CI)	54 000 (49 000 to 59 000)	24 000 (20 000 to 28 000)	18 000 (15 000 to 20 000)	69 000 (60 000 to 78 000)	61 000 (55 000 to 66 000)	62 000 (56 000 to 67 000)	287 000 (273 000 to 301 000)
% (95% CI)	0.29 (0.26 to 0.31)	1.21 (1.00 to 1.41)	0.13 (0.11 to 0.15)	0.27 (0.24 to 0.31)	2.50 (2.26 to 2.71)	0.32 (0.29 to 0.35)	0.35 (0.34 to 0.37)
100%							
Mean (95% CI)	71 000 (66 000 to 77 000)	35 000 (30 000 to 40 000)	27 000 (24 000 to 31 000)	79 000 (70 000 to 89 000)	70 000 (63 000 to 76 000)	100 000 (94 000 to 107 000)	383 000 (368 000 to 399 000)
% (95% CI)	0.38 (0.35 to 0.41)	1.76 (1.51 to 2.01)	0.20 (0.18 to 0.23)	0.31 (0.28 to 0.35)	2.87 (2.59 to 3.12)	0.52 (0.49 to 0.56)	0.47 (0.45 to 0.49)
**Total cases of weight-related diseases averted**							
**Cancer cases averted over lifetime**							
25%							
Mean (95% CI)	670 (−37 to 1400)	−200 (−820 to 410)	−440 (−1100 to 240)	1800 (1000 to 2500)	620 (−29 to 1300)	1000 (350 to 1800)	3500 (1800 to 5200)
% (95% CI)	0.01 (0.00 to 0.02)	−0.01 (−0.03 to 0.01)	−0.01 (−0.02 to 0.00)	0.03 (0.01 to 0.04)	0.02 (0.00 to 0.04)	0.02 (0.01 to 0.03)	0.01 (0.01 to 0.02)
50%							
Mean (95% CI)	1400 (680 to 2100)	1000 (420 to 1600)	360 (−340 to 1100)	2400 (1600 to 3200)	600 (−67 to 1300)	1900 (1100 to 2700)	7700 (6000 to 9400)
% (95% CI)	0.02 (0.01 to 0.03)	0.03 (0.01 to 0.05)	0.01 (−0.01 to 0.02)	0.04 (0.02 to 0.05)	0.02 (0.00 to 0.04)	0.03 (0.02 to 0.05)	0.02 (0.02 to 0.03)
75%							
Mean (95% CI)	2200 (1400 to 2900)	1700 (1100 to 2300)	240 (−450 to 920)	3100 (2400 to 3900)	1700 (1100 to 2300)	2000 (1300 to 2800)	11 000 (9000 to 13 000)
% (95% CI)	0.03 (0.02 to 0.04)	0.06 (0.04 to 0.08)	0.00 (−0.01 to 0.02)	0.05 (0.04 to 0.06)	0.06 (0.04 to 0.07)	0.03 (0.02 to 0.05)	0.04 (0.03 to 0.04)
100%							
Mean (95% CI)	3300 (2600 to 4100)	1700 (1100 to 2300)	860 (130 to 1600)	3600 (2800 to 4400)	2100 (1500 to 2800)	2700 (1900 to 3400)	14 000 (13 000 to 16 000)
% (95% CI)	0.05 (0.04 to 0.06)	0.06 (0.04 to 0.08)	0.02 (0.00 to 0.03)	0.05 (0.04 to 0.06)	0.07 (0.05 to 0.09)	0.05 (0.03 to 0.06)	0.05 (0.04 to 0.05)
**Coronary heart disease events averted over lifetime**							
25%							
Mean (95% CI)	640 (−130 to 1400)	81 (−530 to 690)	−230 (−980 to 530)	6200 (5000 to 7300)	2300 (1400 to 3300)	3100 (2000 to 4200)	12 000 (9900 to 14 000)
% (95% CI)	0.03 (−0.01 to 0.08)	0.01 (−0.06 to 0.08)	−0.01 (−0.06 to 0.03)	0.54 (0.44 to 0.64)	0.28 (0.17 to 0.40)	0.08 (0.05 to 0.11)	0.12 (0.10 to 0.14)
50%							
Mean (95% CI)	1530 (790 to 2300)	1700 (1000 to 2300)	630 (−95 to 1400)	9000 (7700 to 10 000)	4700 (3700 to 5700)	4800 (3700 to 5900)	22 000 (20 000 to 25 000)
% (95% CI)	0.08 (0.04 to 0.12)	0.20 (0.12 to 0.28)	0.04 (−0.01 to 0.09)	0.79 (0.67 to 0.87)	0.56 (0.44 to 0.68)	0.13 (0.10 to 0.16)	0.22 (0.20 to 0.25)
75%							
Mean (95% CI)	3000 (2200 to 3800)	2700 (2000 to 3400)	650 (−71 to 1400)	14 000 (12 800 to 15 000)	8000 (7000 to 9000)	6900 (5800 to 7900)	35 000 (33 000 to 38 000)
% (95% CI)	0.16 (0.12 to 0.21)	0.32 (0.24 to 0.41)	0.04 (0.00 to 0.09)	1.22 (1.12 to 1.31)	0.96 (0.84 to 1.08)	0.18 (0.15 to 0.21)	0.35 (0.33 to 0.38)
100%							
Mean (95% CI)	3000 (2300 to 3800)	3300 (2600 to 3900)	1700 (1000 to 2500)	18 000 (16 000 to 19 000)	8100 (7000 to 9000)	9400 (8300 to 11 000)	43 000 (41 000 to 45 000)
% (95% CI)	0.16 (0.12 to 0.21)	0.40 (0.31 to 0.47)	0.11 (0.06 to 0.16)	1.53 (1.40 to 1.66)	0.97 (0.84 to 1.08)	0.25 (0.22 to 0.29)	0.43 (0.41 to 0.45)
**Diabetes cases averted over lifetime**							
25%							
Mean (95% CI)	1000 (320 to 1800)	−62 (−690 to 570)	390 (−310 to 1100)	6200 (5400 to 7000)	900 (220 to 1600)	2900 (2200 to 3700)	11 000 (9600 to 13 000)
% (95% CI)	0.03 (0.01 to 0.05)	0.00 (−0.05 to 0.04)	0.01 (−0.01 to 0.04)	0.15 (0.13 to 0.17)	0.06 (0.01 to 0.11)	0.09 (0.07 to 0.11)	0.07 (0.06 to 0.08)
50%							
Mean (95% CI)	3600 (2800 to 4300)	2500 (1900 to 3200)	970 (260 to 1700)	8800 (7900 to 9700)	4800 (4200 to 5500)	4500 (3800 to 5300)	25 000 (23 000 to 27 000)
% (95% CI)	0.11 (0.08 to 0.13)	0.16 (0.13 to 0.21)	0.03 (0.01 to 0.06)	0.22 (0.19 to 0.24)	0.32 (0.28 to 0.36)	0.13 (0.11 to 0.16)	0.15 (0.14 to 0.16)
75%							
Mean (95% CI)	4600 (3800 to 5400)	4100 (3500 to 4800)	1200 (480 to 1900)	12 000 (12 000 to 13 000)	7000 (6300 to 7700)	5800 (5000 to 6600)	35,00 (33 000 to 37 000)
% (95% CI)	0.14 (0.11 to 0.16)	0.27 (0.23 to 0.32)	0.04 (0.02 to 0.07)	0.30 (0.30 to 0.32)	0.46 (0.42 to 0.51)	0.17 (0.15 to 0.20)	0.21 (0.20 to 0.22)
100%							
Mean (95% CI)	5100 (4300 to 5800)	5500 (4800 to 6200)	1600 (900 to 2400)	16 000 (15 000 to 17 000)	7800 (7000 to 8500)	8300 (7500 to 9100)	44 000 (42 000 to 46 000)
% (95% CI)	0.15 (0.13 to 0.17)	0.36 (0.32 to 0.41)	0.06 (0.03 to 0.09)	0.39 (0.37 to 0.42)	0.51 (0.46 to 0.56)	0.25 (0.22 to 0.27)	0.26 (0.25 to 0.28)
**Stroke cases averted over lifetime**							
25%							
Mean (95% CI)	−250 (−660 to 160)	−190 (−530 to 150)	50 (−340 to 440)	190 (−570 to 940)	−19 (−640 to 600)	−21 (−680 to 640)	−250 (−1600 to 1100)
% (95% CI)	−0.05 (−0.14 to 0.03)	−0.09 (−0.25 to 0.07)	0.01 (−0.08 to 0.11)	0.01 (−0.04 to 0.06)	−0.01 (−0.30 to 0.28)	0.00 (−0.06 to 0.05)	−0.01 (−0.04 to 0.03)
50%							
Mean (95% CI)	−53 (−430 to 330)	7 (−320 to 330)	260 (−130 to 650)	280 (−400 to 960)	330 (−230 to 890)	−150 (−850 to 540)	670 (−620 to 2000)
% (95% CI)	−0.01 (−0.09 to 0.07)	0.00 (−0.15 to 0.16)	0.06 (−0.03 to 0.16)	0.02 (−0.03 to 0.07)	0.16 (−0.11 to 0.42)	−0.01 (−0.07 to 0.04)	0.02 (−0.02 to 0.05)
75%							
Mean (95% CI)	300 (−97 to 700)	−310 (−650 to 30)	220 (−170 to 600)	−210 (−930 to 510)	−360 (−990 to 260)	−190 (−860 to 490)	−550 (−1900 to 790)
% (95% CI)	0.06 (−0.02 to 0.15)	−0.15 (−0.31 to 0.01)	0.05 (−0.04 to 0.15)	−0.01 (−0.06 to 0.04)	−0.17 (−0.47 to 0.12)	−0.02 (−0.07 to 0.04)	−0.01 (−0.05 to 0.02)
100%							
Mean (95% CI)	230 (−160 to 630)	26 (−330 to 380)	−230 (−620 to 160)	−90 (−790 to 610)	−130 (−670 to 420)	1300 (580 to 1900)	1000 (−220 to 2400)
% (95% CI)	0.05 (−0.03 to 0.13)	0.01 (−0.16 to 0.18)	−0.06 (−0.15 to 0.04)	−0.01 (−0.05 to 0.04)	−0.06 (−0.31 to 0.20)	0.11 (0.05 to 0.15)	0.03 (−0.01 to 0.06)
**Weight-related disease-associated deaths**							
**Cancer-associated deaths averted over lifetime**							
25%							
Mean (95% CI)	1200 (450 to 1900)	310 (−300 to 930)	100 (−590 to 780)	1390 (650 to 2100)	860 (270 to 1460)	660 (−50 to 1370)	4500 (2800 to 6200)
% (95% CI)	0.04 (0.02 to 0.07)	0.02 (−0.02 to 0.07)	0.00 (−0.03 to 0.03)	0.05 (0.02 to 0.07)	0.07 (0.02 to 0.12)	0.03 (0.00 to 0.06)	0.04 (0.02 to 0.05)
50%							
Mean (95% CI)	1800 (1000 to 2500)	1100 (500 to 1700)	300 (−370 to 980)	2500 (1700 to 3200)	1500 (870 to 2200)	1300 (660 to 2000)	8500 (6800 to 10 000)
% (95% CI)	0.06 (0.04 to 0.09)	0.09 (0.04 to 0.14)	0.01 (−0.02 to 0.04)	0.09 (0.06 to 0.11)	0.12 (0.07 to 0.18)	0.05 (0.03 to 0.08)	0.07 (0.05 to 0.08)
75%							
Mean (95% CI)	2300 (1600 to 3000)	2300 (1700 to 2900)	670 (−20 to 1400)	4000 (3200 to 4700)	2100 (1500 to 2700)	2100 (1400 to 2800)	13 000 (12 000 to 15 000)
% (95% CI)	0.08 (0.06 to 0.11)	0.18 (0.14 to 0.23)	0.03 (0.00 to 0.06)	0.14 (0.11 to 0.17)	0.17 (0.12 to 0.22)	0.09 (0.06 to 0.12)	0.10 (0.09 to 0.12)
100%							
Mean (95% CI)	3000 (2200 to 3700)	2900 (2300 to 3500)	800 (100 to 1500)	4100 (3400 to 4800)	2400 (1700 to 3000)	2400 (1700 to 3100)	16 000 (14 000 to 17 000)
% (95% CI)	0.11 (0.08 to 0.13)	0.23 (0.18 to 0.28)	0.03 (0.00 to 0.06)	0.14 (0.12 to 0.17)	0.19 (0.14 to 0.24)	0.10 (0.07 to 0.13)	0.12 (0.11 to 0.13)
**Coronary heart disease–associated deaths averted over lifetime**							
25%							
Mean (95% CI)	440 (60 to 800)	−50 (−370 to 270)	200 (−170 to 560)	2900 (2400 to 3400)	320 (−110 to 740)	1100 (660 to 1600)	4900 (3900 to 6000)
% (95% CI)	0.07 (0.01 to 0.13)	−0.02 (−0.14 to 0.10)	0.04 (−0.03 to 0.11)	0.25 (0.21 to 0.30)	0.12 (−0.04 to 0.28)	0.12 (0.07 to 0.17)	0.13 (0.11 to 0.16)
50%							
Mean (95% CI)	800 (450 to 1200)	560 (240 to 880)	120 (−210 to 460)	3700 (3100 to 4200)	2200 (1700 to 2600)	1900 (1400 to 2400)	9200 (8100 to 10 000)
% (95% CI)	0.13 (0.08 to 0.20)	0.21 (0.09 to 0.33)	0.02 (−0.04 to 0.09)	0.32 (0.27 to 0.37)	0.82 (0.63 to 0.97)	0.20 (0.15 to 0.26)	0.25 (0.22 to 0.27)
75%							
Mean (95% CI)	1500 (1100 to 1900)	1300 (970 to 1600)	440 (70 to 810)	5800 (5300 to 6300)	3170 (2700 to 3600)	2200 (1700 to 2700)	14 000 (13 000 to 15 000)
% (95% CI)	0.25 (0.19 to 0.32)	0.48 (0.36 to 0.60)	0.09 (0.01 to 0.16)	0.51 (0.46 to 0.55)	1.18 (1.01 to 1.34)	0.24 (0.18 to 0.29)	0.38 (0.35 to 0.41)
100%							
Mean (95% CI)	2000 (1600 to 2400)	1800 (1500 to 2100)	590 (240 to 950)	6000 (5500 to 6600)	3900 (3400 to 4300)	3700 (3200 to 4200)	18 000 (17 000 to 19 000)
% (95% CI)	0.34 (0.27 to 0.40)	0.67 (0.56 to 0.78)	0.12 (0.05 to 0.19)	0.52 (0.48 to 0.58)	1.45 (1.27 to 1.60)	0.40 (0.34 to 0.45)	0.49 (0.46 to 0.51)
**Diabetes-associated deaths averted over lifetime**							
25%							
Mean (95% CI)	60 (−40 to 160)	60 (−30 to 140)	40 (−50 to 140)	160 (30 to 290)	180 (80 to 280)	150 (40 to 260)	640 (390 to 900)
% (95% CI)	0.14 (−0.09 to 0.38)	0.31 (−0.16 to 0.73)	0.12 (−0.14 to 0.40)	0.28 (0.05 to 0.51)	0.94 (0.42 to 1.47)	0.33 (0.09 to 0.57)	0.29 (0.18 to 0.41)
50%							
Mean (95% CI)	180 (81 to 280)	89 (6 to 170)	54 (−45 to 150)	370 (250 to 500)	240 (140 to 330)	65 (−50 to 180)	1000 (740 to 1200)
% (95% CI)	0.43 (0.19 to 0.66)	0.47 (0.03 to 0.89)	0.16 (−0.13 to 0.43)	0.65 (0.44 to 0.88)	1.26 (0.73 to 1.73)	0.14 (−0.11 to 0.40)	0.46 (0.34 to 0.55)
75%							
Mean (95% CI)	69 (−30 to 170)	175 (92 to 260)	19 (−76 to 110)	490 (360 to 620)	330 (230 to 430)	210 (100 to 330)	1300 (1000 to 1600)
% (95% CI)	0.16 (−0.07 to 0.40)	0.92 (0.48 to 1.36)	0.05 (−0.22 to 0.32)	0.86 (0.63 to 1.09)	1.73 (1.20 to 2.25)	0.46 (0.22 to 0.73)	0.60 (0.46 to 0.74)
100%							
Mean (95% CI)	150 (47 to 240)	170 (86 to 250)	82 (−16 to 180)	560 (420 to 690)	340 (240 to 430)	280 (170 to 400)	1600 (1300 to 1800)
% (95% CI)	0.35 (0.11 to 0.57)	0.89 (0.45 to 1.31)	0.24 (−0.05 to 0.52)	0.99 (0.74 to 1.22)	1.78 (1.26 to 2.25)	0.62 (0.37 to 0.88)	0.74 (0.60 to 0.83)

^a^ Some mean values are negative due to variation in the model causing nonsignificant increases when disparities are reduced.

Knowing that it may be ambitious to fully eliminate PA disparities among SES groups, Table 2 summarizes the estimated effect of reducing the disparity by different degrees. For example, closing the SES disparity gap by 25% results in approximately 27 000 (95% CI, 24 000-30 000) fewer total cases of weight-related diseases over the cohort’s lifetime. This further increases to 55 000 (95% CI, 52 000-59 000) fewer cases of weight-related diseases when closing the gap by 50% and up to 81 000 (95% CI, 78 000-85 000) fewer cases when closing it by 75%.

However, different age and sex groups do not have equal health benefits from reducing disparities, as different groups have different disparities in PA levels, overweight/obesity prevalence, and population sizes. For example, adolescent boys aged 14 to 17 years at 100% to 199% FPL experience the greatest reduction in overweight and obesity with 63 000 fewer cases and 12 000 (95% CI, 11 000-13 000) weight-related disease cases averted when eliminating the disparity. On the other hand, adolescent girls aged 14 to 17 years at 200% to 399% FPL experience the least amount of health benefits from reducing the disparity, with 2700 fewer cases of overweight and obesity and 410 (95% CI, −500 to 1300) fewer cases of weight-related diseases.

### Estimated Economic Effect of Reducing Disparities in PA Levels Among Youth in Different SES Groups Nationwide

**Figure 2.**
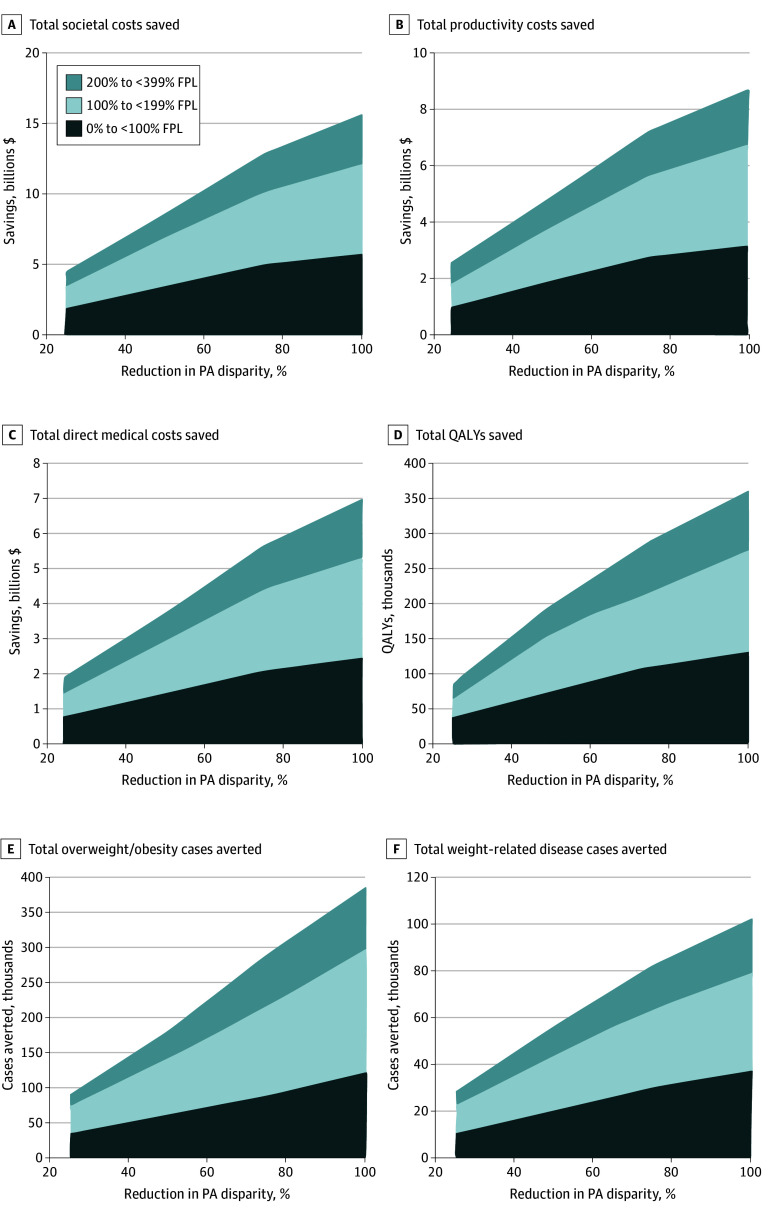
Economic and Clinical Outcomes of Reducing Physical Activity (PA) Disparities Among US Youth by Socioeconomic Status Group All reductions in PA disparity are compared with the highest PA level for each age and sex group. FPL indicates federal poverty level; QALYs, quality-adjusted life-years.

Figure 2 shows the resulting savings from the societal perspective for the different age and sex groups. The improved physical health outcomes achieved by eliminating the disparity in PA levels among SES groups also results in more than $15.60 (95% CI, $15.01-$16.10) billion in cost savings over the cohort’s lifetime, of which 44% is direct medical cost savings and 56% is productivity losses averted.

The savings generated vary across the SES, age, and sex groups. For example, eliminating the disparity saves $847.77 (95% CI, $756.93-$939.61) million in societal costs for girls 11 to 13 years old at 100% to 199% FPL (societal perspective) but saves only $41.48 (95% CI, −$91.15 to $170.41) million for adolescent girls aged 14 to 17 years at 200% to 399% FPL. Similar to the health benefits, these cost-savings values are affected by the size of the disparity between groups, the starting overweight/obesity prevalence in each group, and the group’s size.

If the US reduces the SES disparity in each age and sex group (compared with the SES group with the highest PA levels in that age and sex category) by just 25%, this still saves $4.34 (95% CI, $3.87-$4.80) billion in societal costs. This increases to $8.17 (95% CI, $7.12-$9.22) billion and $12.75 (95% CI, $12.27-$13.24) billion in societal costs when reducing disparities by 50% and 75%, respectively. Cost savings increase approximately linearly with the disparity reduction (eg, decreasing by $153.4 million per 1% in disparity reduction).

### Estimated Effect of Varying the Effect PA Has on the Weight of Individuals in Lower SES Groups

Even when increasing compensatory calorie consumption to 25% and 50% among lower SES groups (ie, <100% FPL and 100%-199% FPL), a 100% reduction in PA disparities would still lead to absolute decreases in overweight and obesity prevalence by 0.456% (95% CI, 0.452%-0.461%) and 0.387% (95% CI, 0.383%-0.391%), saving $13.53 (95% CI, $13.04-$14.02) and $10.69 (95% CI, $10.21-$11.18) billion in societal cost savings, respectively. Decreasing total caloric consumption by 2% would save $55.49 (95% CI, $54.97-$56.01) billion in societal costs.

### Estimated Effect of Varying the Amount of PA on Days Not Getting 60 Minutes

When varying the minutes of PA on days youth do not get at least 60 minutes down to 0 minutes per day, there are decreases in overweight and obesity prevalence by an absolute 1.77% (95% CI, 1.76%-1.78%), saving $29.39 (95% CI, $28.80- $29.99) billion in total societal costs. If getting 15 minutes per day, eliminating the disparities results in an absolute decrease in overweight/obesity prevalence of 1.37% (95% CI, 1.36%-1.38%), saving $24.20 (95% CI, $23.59-$24.82) billion in societal costs. With 45 minutes per day of PA, eliminating disparities still results in an absolute overweight/obesity prevalence decrease of 0.41% (95% CI, 0.40%-0.41%), saving $8.48 (95% CI, $7.91-$9.05) billion in societal costs.

## Discussion

Simulation results show that eliminating SES disparities in PA among youth in the US could generate recurrent savings ($15.60 billion for each new cohort of 6- to 17-year-olds), which would be 4.7 to 8.2 times the cost of currently available school-based PA interventions and elementary school active PE policies, based on published estimates of $0.32 to $0.42 per metabolic equivalent of hours per day per person (in 2023 values).^28,29,30^ Of course, this does not necessarily mean that the US can and should spend close to $15 billion on a recurring basis to decrease PA disparities. State and local governments already spend $12 billion (2021 US dollars) per year in the US on building new parks and recreation areas (ie, capital projects).^31^ It is not clear how much more investment would be needed to expand these efforts to adequately increase access to lower-socioeconomic communities and how much effect this expanded access would have on youth PA in those communities. Moreover, it is also not clear where such funds would come from and whether they would be shifted from other areas of need.

Nevertheless, it is also important to remember that PA influences health and subsequent costs through other benefits besides weight status (eg, bone density, muscle strengthening, likelihood of anxiety, depression); thus, results may underestimate the potential cost savings of eliminating such disparities. As indicated in the Methods section, because the goal of this study was to determine what would happen if we eliminated or reduced PA disparities, the experiments assumed that any increases in PA would be maintained throughout childhood/adolescence. This would mean that policies or interventions to increase PA among those of lower SES may have to be maintained or even adapted from age 6 through 17 years. It cannot be assumed that an increase in PA at an earlier age will be sustainable throughout a child’s subsequent years (eg, evidence suggests that many youth drop out of sports participation at age 11^32,33^). Thus, interventions designed to increase sports and PA participation among lower-income youth will need to be affordable, consider and address transportation needs, invest in recreation spaces, train coaches, address injury prevention, and ensure that youth have fun to overcome them dropping out of sports and PA.^1,34,35,36^

Additionally, it is important to note that increases in resources may not overcome all barriers to changing weight across different SES groups. The sensitivity analyses attempted to account for some potential differences among various SES groups in the relationship between PA and overweight and obesity (eg, changes in compensatory eating and dietary habits). However, there are some other potential differences such as access to health care and alcohol and drug use.

To our knowledge, this is the first study of its kind to quantify the health and economic benefits that can be achieved by eliminating PA disparities among youth in different SES groups. While previous studies have quantified the consequences of insufficient PA and its association with increased health-related problems and costs,^37,38,39^ as well as examined specific PA interventions and found that they often result in uneven gains among SES groups,^40^ they have not yet attempted to quantify the potential long-term benefits of programs targeting these disparities. The present study is critical to understanding the scale of potential benefits of programs addressing these inequalities and guiding the prioritization of efforts to reduce SES disparities in youth PA.

To actually address these disparities, there are a number of policies and interventions that policymakers can consider. For example, allocating funding to school physical education systems so underresourced children can engage in quality PA, investing in the development of PA facilities in lower-income neighborhoods (eg, green spaces, recreational gyms), or by increasing neighborhood maintenance of existing PA resources to improve neighborhood perceptions and the physical environment.^34^ Additionally, transit-oriented development and mixed-use zoning can help connect individuals to PA infrastructure as well as encourage active transport (eg, walking, biking, using wheelchair).^35^ Policymakers and community leaders can also develop joint-use agreements that allow youth to use existing infrastructure (eg, schools, playgrounds, pools) after regularly scheduled programming.^36^ Future studies can further examine the drivers of PA disparities by SES and quantify the effectiveness of these policies and programs in different social and built-environment contexts to inform the design of effective interventions.

### Limitations

All models, by definition, are simplifications of reality and cannot account for all factors. To remain conservative about the potential positive effect of PA, this study focused on BMI and weight-related chronic health conditions and did not include other potential outcomes (eg, osteoporosis). The model also did not include the potential effect of PA on mental health or the other potential benefits of PA and sports for youth (eg, improved academic performance, social skills, emotional regulation, mood).^41,42^ Moreover, changes in PA disparities by SES likely have differential effects across racial and ethnic populations given long-standing structural discrimination and racism that have led to racialized patterns of socioeconomic resources in the US. Due to limited available data on PA levels by SES, age, and sex across different racial and ethnic groups in the US, this study was unable to examine the intersectional effects of SES, race, and ethnicity on the study outcomes; this can be examined in future studies.

## Conclusions

This study shows that eliminating or reducing disparities in youth PA between SES groups and subsequent reductions in cardiometabolic and cancer events could save the US $4.34 to $15.60 billion in direct medical costs and productivity losses over the lifetimes of each 6- to 17-year-old cohort. Quantifying the potential savings from eliminating and reducing PA disparities can help policymakers, health care systems, schools, funders, sports organizations, and other businesses understand the magnitude of potential savings and, thus, how to best prioritize investments toward addressing these disparities.

## Notes

Supplement 1. **eTable 1.** CEOM Table of Inputs **eTable 2. **Transition probabilities for adults Age ≤45 years old **eTable 3. **Transition probabilities for adults Age >45 years old
eReferences

Supplement 2. Data Sharing Statement
