# Supplementary material for: Health and Economic Value of Eliminating Socioeconomic Disparities in US Youth Physical Activity
Source: JAMA Health Forum. 2024 Mar 15;5(3):e240088. doi: 10.1001/jamahealthforum.2024.0088 (PMC10943408; doi:10.1001/jamahealthforum.2024.0088)
Supplement: Supplement 1. — eTable 1. CEOM Table of Inputs eTable 2. Transition probabilities for adults Age ≤45 years old eTable 3. Transition probabilities for adults Age >45 years old eReferences [file jamahealthforum-e240088-s001.pdf]

Supplemental Online Content

Powell-Wiley TM, Martinez MF, Heneghan J, et al. Health and economic value of eliminating socioeconomic disparities in US youth physical activity. *JAMA Health Forum*. 2024;5(3):e240088. doi:10.1001/jamahealthforum.2024.0088

eTable 1. CEOM Table of Inputs
eTable 2. Transition probabilities for adults Age ≤45 years old
eTable 3. Transition probabilities for adults Age >45 years old
eReferences

This supplemental material has been provided by the authors to give readers additional information about their work.

© 2024 Powell-Wiley TM et al. *JAMA Health Forum.*

eTable 1. CEOM Table of Inputs

Cost Values (2023 Dollars)	Distribution type	Mean	Range or standard deviation	Source
Chronic Health State 1		$611.25		(1)
Chronic Health State 2		$1,381.15		(1)
Normal Weight Chronic Health State 3		$2,066.19		(1)
Overweight Chronic Health State 3		$2,527.39		(1)
Obese Chronic Health State 3		$4,833.40		(1)
Normal Weight Chronic Health State 4		$3,574.01		(1)
Overweight Chronic Health State 4		$4,496.42		(1)
Obese Chronic Health State 4		$9,108.45		(1)
Heart Disease: Age 18-44	Gamma	$5,859.21	$2,694.88	(2)
Heart Disease: Age 45- 65	Gamma	$7,888.34	$1,329.05	(2)
Heart Disease: Age 65+	Gamma	$5,394.44	$435.16	(2)
Diabetes Mellitus: Age 18-44	Gamma	$5,791.43	$1,196.88	(2)
Diabetes Mellitus: Age 44-65	Gamma	$5,674.34	$389.39	(2)
Diabetes Mellitus: Age 65+	Gamma	$4,524.74	$229.81	(2)
Hypertension: Age 18-44	Gamma	$810.58	$316.67	(2)
Hypertension: Age 44-65	Gamma	$668.63	$77.20	(2)
Hypertension: Age 65+	Gamma	$834.39	$87.06	(2)
Diabetic Nephropathy	Triangular	$1,123.50	$1,011 - $1,236	(3)
ESRD-Initial year	Triangular	$86,805.14	$78,125 - $95,486	(4)
Diabetic Neuropathy	Triangular	$15,824.49	$14,242 - $17,407	(5)
Diabetic Retinopathy	Triangular	$4,891.86	$4,403 - $5,381	(6)
Blindness	Triangular	$8,977.36	$8,080 - $9,875	(6)
Cerebrovascular Disease, including Stroke: Age 18-64	Gamma	$11,183.71	$4,326.88	(2)
Cerebrovascular Disease, including Stroke: Age 65+	Gamma	$8,119.39	$1,374.24	(2)
Breast Cancer: First Year, Females		$39,474.44		(7)
Breast Cancer: After First Year, Females		$4,777.84		(7)
Breast Cancer: Last Year, Females		$86,115.63		(7)
Cervical Cancer: First Year, Females		$64,160.12		(7)
Cervical Cancer: After First Year, Females		$4,322.83		(7)
Cervical Cancer: Last Year, Females		$106,592.13		(7)
Colorectal Cancer, First Year		$73,147.04		(7)
Colorectal Cancer: After First Year		$7,053.01		(7)
Colorectal Cancer: Last Year		$121,835.89		(7)
Esophageal Cancer, First Year		$99,994.14		(7)
Esophageal Cancer: After First Year		$11,603.34		(7)
Esophageal Cancer: Last Year		$132,188.06		(7)
Renal Cancer: First Year		$47,437.46		(7)
Renal Cancer: After First Year		$11,375.94		(7)
Renal Cancer: Last Year		$117,740.57		(7)
Cost Values (2023 Dollars)	Distribution type	Mean	Range or standard deviation	Source
Pancreatic Cancer: First Year		$124,224.81		(7)
Pancreatic Cancer: After First Year		$24,344.43		(7)
Pancreatic Cancer: Last Year		$142,995.02		(7)
Stomach Cancer: First Year		$90,210.95		(7)
Stomach Cancer: After First Year		$10,465.81		(7)

© 2024 Powell-Wiley TM et al. *JAMA Health Forum.*

Stomach Cancer: Last Year		$135,259.50		(7)
Uterine Cancer: First Year, Females		$42,773.38		(7)
Uterine Cancer: After First Year, Females		$3,299.05		(7)
Uterine Cancer: Last Year, Females		$103,520.69		(7)
Prostate cancer - First Year, Males		$31,056.18		(7)
Prostate cancer - After First Year, Males		$3,185.19		(7)
Prostate cancer - Last Year, Males		$87,480.66		(7)
Annual Wages	Triangular	$63,757	$28,160 - $113,599	(8)
**Probability Values**	**Distribution type**	**Mean**	**Range or standard deviation**	**Source**
**Probability of developing coronary heart disease (CHD)**				
At Chronic Health State 2	Triangular	0.01023	0 - 0.04	(9, 10)
At Chronic Health State 3	Triangular	0.01193	0 - 0.047	(9, 10)
At Chronic Health State 4	Triangular	0.01813	0 - 0.063	(9, 10)
Multiplier for increased probability of CHD for individual with overweight	Triangular	1.31	1.22 - 1.4	(10)
Multiplier for increased probability of CHD for individual with obesity	Triangular	1.56	1.54 - 1.58	(10)
**Probability of reoccurrence of CHD**				
At Chronic Health State 2	Triangular	0.0427	0 - 0.1	(11)
At Chronic Health State 3	Triangular	0.0347	0 - 0.057	(11)
At Chronic Health State 4	Triangular	0.0397	0 - 0.074	(11)
**Probability of death due to CHD**				
At Chronic Health State 2	Triangular	0.01023	0 - 0.04	(9, 10)
At Chronic Health State 3	Triangular	0.01193	0 - 0.047	(9, 10)
At Chronic Health State 4	Triangular	0.037	0.0007 - 0.113	(9, 10)
Multiplier for increased probability of death for individual with overweight	Triangular	1.175	0.98 - 1.37	(9, 10)
Multiplier for increased probability of death for individual with obesity	Triangular	1.375	1.3 - 1.45	(9, 10)
**Probability of developing Stroke**				
At Chronic Health State 0	Uniform	0.06	0 - 0.012	(9, 10, 12)
At Chronic Health State 1	Uniform	0.075	0 - 0.015	(9, 10, 12)
At Chronic Health State 2	Uniform	0.085	0 - 0.017	(9, 10, 12)
At Chronic Health State 3	Uniform	0.075	0 - 0.015	(9, 10, 12)
At Chronic Health State 4	Uniform	0.014	0 - 0.028	(9, 10, 12)
Multiplier for increased probability of stroke for individual with overweight	Uniform	1.115	1.06 - 1.17	(13, 14)
Multiplier for increased probability of stroke for individual with obesity	Uniform	1.325	1.23 - 1.42	(13, 14)
Probability of reoccurrence of Stroke at first year	Uniform	0.1287	0.0924 - 0.165	(15)
Probability of reoccurrence of Stroke after first year	Uniform	0.0443	0.0318 - 0.0567	(15)
**Probability of death due to stroke**				
In the first year of developing stroke	Uniform	0.188	0.135 - 0.241	(15)
**Probability Values**	**Distribution type**	**Mean**	**Range or standard deviation**	**Source**
After First year of developing stroke	Uniform	0.083	0.0596 - 0.1064	(15)
**Probabilities associated with diabetes complications**				
Probability of developing Diabetic nephropathy by years of having T2DM	Uniform	0.14	0 - 0.28	(16)
Probability of developing ESRD		0.0082		(17)

Probability of death from ESRD (by age)	Uniform	0.16	0.081 - 0.239	(18)
Probability of developing Diabetic neuropathy by years of having T2DM	Uniform	0.36	0 - 0.72	(16)
Probability of Diabetic neuropathy by years of having T2DM	Uniform	0.14	0 - 0.28	(16)
Probability of developing blindness	Uniform	0.4	0 - 0.8	(19, 20)
**Probabilities of developing Cancer for females**				
Breast	Uniform	0.0096	0 - 0.0191	(21, 22)
Cervical	Uniform	0.00035	0 - 0.0007	(21, 22)
Colorectal	Uniform	0.0033	0 - 0.0066	(21, 22)
Esophageal	Uniform	0.0002	0 - 0.0004	(21, 22)
Renal	Uniform	0.0009	0 - 0.0018	(21, 22)
Pancreatic	Uniform	0.00125	0 - 0.0025	(21, 22)
Stomach	Uniform	0.0005	0 - 0.001	(21, 22)
Uterine	Uniform	0.0022	0 - 0.0044	(21, 22)
**Probabilities of developing Cancer for males**				
Colorectal	Uniform	0.0036	0 - 0.0071	(21, 22)
Esophageal	Uniform	0.00065	0 - 0.0013	(21, 22)
Renal	Uniform	0.00155	0 - 0.0031	(21, 22)
Pancreatic	Uniform	0.00125	0 - 0.0025	(21, 22)
Prostate	Uniform	0.0142	0 - 0.0284	(21, 22)
Stomach	Uniform	0.00085	0 - 0.0017	(21, 22)
**Probabilities of death due to Cancer for females**				
Breast	Uniform	0.034	0.008 - 0.06	(22)
Cervical	Uniform	0.0445	0.006 - 0.083	(22)
Colorectal	Uniform	0.066	0.027 - 0.105	(22)
Esophageal	Uniform	0.2305	0.035 - 0.426	(22)
Renal	Uniform	0.034	0.011 - 0.057	(22)
Pancreatic	Uniform	0.161	0.033 - 0.289	(22)
Stomach	Uniform	0.172	0.03 - 0.314	(22)
Uterine	Uniform	0.0275	0.007 - 0.048	(22)
**Probabilities of death due to Cancer for males**				
Colorectal	Uniform	0.089	0.016 - 0.162	(22)
Esophageal	Uniform	0.2135	0.018 - 0.409	(22)
Renal	Uniform	0.0385	0.008 - 0.069	(22)
Pancreatic	Uniform	0.1765	0.018 - 0.335	(22)
Prostate	Uniform	0.033	0.015 - 0.051	(22)
Stomach	Uniform	0.1395	0.015 - 0.264	(22)
**Utility Values**	**Distribution type**	**Mean**	**Range or standard deviation**	**Source**
Stroke	Beta	0.6	0.09	(23-69)
CHD	Beta	0.73	0.1	(23-69)
Diabetic nephropathy	Beta	0.74	0.09	(23-69)
Diabetic neuropathy	Beta	0.65	0.04	(23-69)
Diabetic retinopathy	Beta	0.78	0.04	(23-69)
ESRD	Beta	0.63	0.03	(23-69)
Blindness	Beta	0.52	0.06	(23-69)
**Utility Values**	**Distribution type**	**Mean**	**Range or standard deviation**	**Source**
Renal Cancer	Beta	0.7	0.06	(23-69)
Cervical Cancer	Beta	0.63	0.11	(23-69)
Pancreatic Cancer	Beta	0.66	0.08	(23-69)
Gastric Cancer	Beta	0.52	0.08	(23-69)
Hypertension	Beta	0.97	0.01	(23-69)

© 2024 Powell-Wiley TM et al. *JAMA Health Forum.*

Prostate	Beta	0.71	0.16	(23-69)
DM2	Beta	0.85	0.08	(23-69)
Breast Cancer, First Year	Beta	0.66	0.06	(23-69)
Breast Cancer, After First Year	Beta	0.77	0.06	(23-69)
Breast Cancer, Last Year	Beta	0.23	0.001	(23-69)
Colon Cancer, First Year	Beta	0.52	0.12	(23-69)
Colon Cancer, After First Year	Beta	0.83	0.05	(23-69)
Colon Cancer, Last Year	Beta	0.3	0.001	(23-69)
Esophageal Cancer, Early Years	Beta	0.71	0.22	(23-69)
Esophageal Cancer, Last Year	Beta	0.34	0.001	(23-69)
Uterine Cancer, Early Years	Beta	0.69	0.15	(23-69)
Uterine Cancer, Last Year	Beta	0.79	0.11	(23-69)

© 2024 Powell-Wiley TM et al. *JAMA Health Forum.*

eTable 2. Transition probabilities for adults Age ≤45 years old

	N_C HS0	N_C HS1	N_C HS2	N_C HS3	N_C HS4	OW_C HS0	OW_C HS1	OW_C HS2	OW_C HS3	OW_C HS4	OB_C HS0	OB_C HS1	OB_C HS2	OB_C HS3	OB_C HS4
**N_CHS0**	0.932	0.016	0.001	0	0	0.048	0.002	0	0	0	0	0	0	0	0
**N_CHS1**	0.023	0.9	0.007	0.007	0	0.002	0.051	0.002	0.002	0	0.001	0.001	0.003	0	0
**N_CHS2**	0.002	0.015	0.811	0.012	0.007	0	0	0.067	0.046	0.007	0	0	0.023	0.002	0.001
**N_CHS3**	0	0	0.024	0.844	0.002	0	0	0.046	0.067	0.003	0	0	0	0.002	0.001
**N_CHS4**	0	0	0	0.058	0.86	0	0	0	0	0.046	0	0	0	0	0.026
**OW_CHS0**	0.008	0.001	0	0	0	0.93	0.019	0.001	0	0	0.028	0.003	0.001	0	0
**OW_CHS1**	0.001	0.009	0.003	0.002	0.001	0.018	0.90	0.014	0.008	0	0.004	0.027	0.003	0.003	0
**OW_CHS2**	0	0.003	0.019	0.007	0	0.027	0.049	0.807	0.012	0.011	0	0.004	0.039	0.008	0.004
**OW_CHS3**	0	0	0	0.009	0	0	0	0.025	0.879	0	0	0	0.041	0.041	0
**OW_CHS4**	0	0	0	0.002	0.022	0	0	0	0.005	0.936	0	0	0	0	0.03
**OB_CHS0**	0	0	0	0	0	0.006	0	0	0	0	0.94	0.04	0.002	0.001	0.001
**OB_CHS1**	0	0.001	0	0	0	0.002	0.002	0	0	0	0.016	0.94	0.025	0.008	0.002
**OB_CHS2**	0	0.002	0	0	0	0	0.008	0.002	0.001	0	0.025	0.037	0.88	0.028	0.015
**OB_CHS3**	0	0	0.001	0.006	0	0	0	0.004	0.01	0.003	0	0	0.046	0.921	0.008
**OB_CHS4**	0	0	0	0	0.001	0	0	0	0	0.012	0	0	0	0	0.986

Note: N = Normal Weight; OW = Overweight; OB = Obesity

© 2024 Powell-Wiley TM et al. *JAMA Health Forum.*

eTable 3. Transition probabilities for adults Age >45 years old

	N_C HS0	N_C HS1	N_C HS2	N_C HS3	N_C HS4	OW_C HS0	OW_C HS1	OW_C HS2	OW_C HS3	OW_C HS4	OB_C HS0	OB_C HS1	OB_C HS2	OB_C HS3	OB_C HS4
**N_CHS0**	0.865	0.102	0.001	0	0	0.012	0.011	0.002	0	0	0.001	0.001	0	0	0
**N_CHS1**	0.034	0.902	0.024	0.014	0	0.006	0.012	0.002	0.002	0	0.001	0	0.002	0	0
**N_CHS2**	0.036	0.053	0.869	0.015	0.004	0	0	0.015	0.002	0	0	0	0	0.001	0
**N_CHS3**	0	0	0.008	0.959	0.008	0	0	0	0.018	0.001	0	0	0	0	0
**N_CHS4**	0	0	0.007	0.011	0.948	0	0	0	0	0.018	0	0	0	0	0.002
**OW_CHS0**	0.008	0.004	0	0	0	0.876	0.069	0.013	0	0	0.013	0.014	0.001	0	0
**OW_CHS1**	0.004	0.004	0.003	0	0	0.023	0.912	0.027	0.014	0	0.002	0.007	0.002	0	0
**OW_CHS2**	0	0.007	0.004	0.001	0	0.003	0.039	0.90	0.019	0.006	0	0.002	0.009	0.001	0.001
**OW_CHS3**	0	0	0	0.005	0	0	0	0.013	0.957	0.01	0	0	0.001	0.009	0
**OW_CHS4**	0	0	0	0.001	0.007	0	0	0	0.009	0.962	0	0	0	0	0.01
**OB_CHS0**	0.001	0	0	0	0	0.007	0	0	0	0	0.866	0.079	0.043	0	0.002
**OB_CHS1**	0	0	0	0	0	0.003	0.005	0	0	0	0.016	0.913	0.039	0.02	0
**OB_CHS2**	0	0.001	0	0	0	0	0.001	0.005	0.001	0	0.001	0.032	0.919	0.026	0.011
**OB_CHS3**	0	0	0	0	0	0	0	0.001	0.006	0.001	0	0	0.013	0.959	0.017
**OB_CHS4**	0	0	0	0	0	0	0	0	0	0.008	0	0	0	0	0.984

Note: N = Normal Weight; OW = Overweight; OB = Obesity

© 2024 Powell-Wiley TM et al. *JAMA Health Forum.*

eReferences

1. Agency for Healthcare Research and Quality. Medical Expenditure Panel Survey In: Agency for Healthcare Research and Quality, editor.: Agency for Healthcare Research and Quality,; 2012.
2. Medical Expenditure Panel Survey (MEPS) Household Component (HC) 2020 [Internet]. Available from: https://datatools.ahrq.gov/meps-hc?type=tab&tab=mepshch3uep.
3. Nichols GA, Vupputuri S, Lau H. Medical care costs associated with progression of diabetic nephropathy. Diabetes Care. 2011;34(11):2374-8.
4. United States Renal Data System. 2022 USRDS Annual Data Report: Epidemiology of kidney disease in the United States. National Institutes of Health, National Institute of Diabetes and Digestive and Kidney Diseases, Bethesda, MD, 2022.
5. Sadosky A, Mardekian J, Parsons B, Hopps M, Bienen EJ, Markman J. Healthcare utilization and costs in diabetes relative to the clinical spectrum of painful diabetic peripheral neuropathy. J Diabetes Complications. 2015;29(2):212-7.
6. NORC at the University of Chicago. Cost of Vision Problems: The Economic Burden of Vision Loss and Eye Disorders in the United States. 2013.
7. National Cancer Institute N, HHS, . Cancer Trends Progress Report. 2022.
8. Bureau of Labor Statistics U.S. Department of Labor. Occupational Employment and Wage Statistics: Bureau of Labor Statistics; 2022 [updated May 2022; cited 2023 May 18]. Available from: http://www.bls.gov/oes/current/oes_nat.htm.
9. D'Agostino Sr RB, Grundy S, Sullivan LM, Wilson P. Validation of the Framingham coronary heart disease prediction scores: results of a multiple ethnic groups investigation. Jama. 2001;286(2):180-7.
10. Wilson PW, D'Agostino RB, Sullivan L, Parise H, Kannel WB. Overweight and obesity as determinants of cardiovascular risk: the Framingham experience. Archives of internal medicine. 2002;162(16):1867-72.
11. Rea TD, Heckbert SR, Kaplan RC, Smith NL, Lemaitre RN, Psaty BM. Smoking status and risk for recurrent coronary events after myocardial infarction. Annals of Internal Medicine. 2002;137(6):494-500.
12. Pender JR, Pories WJ. Epidemiology of obesity in the United States. Gastroenterology Clinics of North America. 2005;34(1):1-7.
13. Rexrode KM, Hennekens CH, Willett WC, Colditz GA, Stampfer MJ, Rich-Edwards JW, et al. A prospective study of body mass index, weight change, and risk of stroke in women. Jama. 1997;277(19):1539-45.
14. Walker SP, Rimm EB, Ascherio A, Kawachi I, Stampfer MJ, Willett WC. Body size and fat distribution as predictors of stroke among US men. American Journal of Epidemiology. 1996;144(12):1143-50.
15. Sacco RL, Shi T, Zamanillo M, Kargman D. Predictors of mortality and recurrence after hospitalized cerebral infarction in an urban community The Northern Manhattan Stroke Study. Neurology. 1994;44(4):626-.
16. Orchard TJ, Dorman JS, Maser RE, Becker DJ, Drash AL, Ellis D, et al. Prevalence of complications in IDDM by sex and duration: Pittsburgh Epidemiology of Diabetes Complications Study II. Diabetes. 1990;39(9):1116-24.
17. Kiberd BA, Clase CM. Cumulative risk for developing end-stage renal disease in the US population. Journal of the American Society of Nephrology. 2002;13(6):1635-44.
18. Saran R, Li Y, Robinson B, Abbott KC, Agodoa L, Ayanian J, et al. US Renal Data System 2015 Annual Data Report: Epidemiology of Kidney Disease in the United States.

© 2024 Powell-Wiley TM et al. *JAMA Health Forum.*

American journal of kidney diseases: the official journal of the National Kidney Foundation. 2016;67(3 Suppl 1):A7.
19. Yau JW, Rogers SL, Kawasaki R, Lamoureux EL, Kowalski JW, Bek T, et al. Global prevalence and major risk factors of diabetic retinopathy. Diabetes care. 2012;35(3):556-64.
20. Klein R, Klein BE, Moss SE, Cruickshanks KJ. Relationship of hyperglycemia to the long-term incidence and progression of diabetic retinopathy. Archives of internal medicine. 1994;154(19):2169-78.
21. Fay MP, Pfeiffer R, Cronin KA, Le C, Feuer EJ. Age-conditional probabilities of developing cancer. Statistics in medicine. 2003;22(11):1837-48.
22. Hayat MJ, Howlader N, Reichman ME, Edwards BK. Cancer statistics, trends, and multiple primary cancer analyses from the Surveillance, Epidemiology, and End Results (SEER) Program. The oncologist. 2007;12(1):20-37.
23. Adarkwah CC, Gandjour A, Akkerman M, Evers S. To treat or not to treat? Cost-effectiveness of ace inhibitors in non-diabetic advanced renal disease - a Dutch perspective. Kidney Blood Press Res. 2013;37(2-3):168-80.
24. Aspinall SL, Smith KJ, Good CB, Zhao X, Stone RA, Tonnu-Mihara IQ, et al. Incremental cost effectiveness of pharmacist-managed erythropoiesis-stimulating agent clinics for non-dialysis-dependent chronic kidney disease patients. Appl Health Econ Health Policy. 2013;11(6):653-60.
25. Athanasakis K, Petrakis I, Karampli E, Vitsou E, Lyras L, Kyriopoulos J. Pregabalin versus gabapentin in the management of peripheral neuropathic pain associated with post-herpetic neuralgia and diabetic neuropathy: a cost effectiveness analysis for the Greek healthcare setting. BMC Neurol. 2013;13:56.
26. Attard CL, Brown S, Alloul K, Moore MJ. Cost-effectiveness of folfirinox for first-line treatment of metastatic pancreatic cancer. Curr Oncol. 2014;21(1):e41-51.
27. Bellows BK, Dahal A, Jiao T, Biskupiak J. A cost-utility analysis of pregabalin versus duloxetine for the treatment of painful diabetic neuropathy. J Pain Palliat Care Pharmacother. 2012;26(2):153-64.
28. Boger PC, Turner D, Roderick P, Patel P. A UK-based cost-utility analysis of radiofrequency ablation or oesophagectomy for the management of high-grade dysplasia in Barrett's oesophagus. Aliment Pharmacol Ther. 2010;32(11-12):1332-42.
29. Borisenko O, Beige J, Lovett EG, Hoppe UC, Bjessmo S. Cost-effectiveness of Barostim therapy for the treatment of resistant hypertension in European settings. J Hypertens. 2014;32(3):681-92.
30. Botteman MF, Meijboom M, Foley I, Stephens JM, Chen YM, Kaura S. Cost-effectiveness of zoledronic acid in the prevention of skeletal-related events in patients with bone metastases secondary to advanced renal cell carcinoma: application to France, Germany, and the United Kingdom. Eur J Health Econ. 2011;12(6):575-88.
31. Bresse X, Goergen C, Prager B, Joura E. Universal vaccination with the quadrivalent HPV vaccine in Austria: impact on virus circulation, public health and cost-effectiveness analysis. Expert Rev Pharmacoecon Outcomes Res. 2014;14(2):269-81.
32. Brown ST, Grima DG, Sauriol L. Cost-effectiveness of insulin glargine versus sitagliptin in insulin-naive patients with type 2 diabetes mellitus. Clin Ther. 2014;36(11):1576-87.
33. Choudhry NK, Patrick AR, Glynn RJ, Avorn J. The cost-effectiveness of C-reactive protein testing and rosuvastatin treatment for patients with normal cholesterol levels. J Am Coll Cardiol. 2011;57(7):784-91.
34. Das A, Ngamruengphong S, Nagendra S, Chak A. Asymptomatic pancreatic cystic neoplasm: a cost-effectiveness analysis of different strategies of management. Gastrointest Endosc. 2009;70(4):690-9 e6.

© 2024 Powell-Wiley TM et al. *JAMA Health Forum.*

35. Dorian P, Kongnakorn T, Phatak H, Rublee DA, Kuznik A, Lanitis T, et al. Cost-effectiveness of apixaban vs. current standard of care for stroke prevention in patients with atrial fibrillation. Eur Heart J. 2014;35(28):1897-906.
36. Folse HJ, Goswami D, Rengarajan B, Budoff M, Kahn R. Clinical- and cost-effectiveness of LDL particle-guided statin therapy: a simulation study. Atherosclerosis. 2014;236(1):154-61.
37. Fonseca T, Clegg J, Caputo G, Norrbacka K, Dilla T, Alvarez M. The cost-effectiveness of exenatide once weekly compared with exenatide twice daily and insulin glargine for the treatment of patients with type two diabetes and body mass index >/=30 kg/m(2) in Spain. J Med Econ. 2013;16(7):926-38.
38. Gladwell D, Henry T, Cook M, Akehurst R. Cost effectiveness of renal denervation therapy for the treatment of resistant hypertension in the UK. Appl Health Econ Health Policy. 2014;12(6):611-22.
39. Gomes M, Aldridge RW, Wylie P, Bell J, Epstein O. Cost-effectiveness analysis of 3-D computerized tomography colonography versus optical colonoscopy for imaging symptomatic gastroenterology patients. Appl Health Econ Health Policy. 2013;11(2):107-17.
40. Green LE, Dinh TA, Hinds DA, Walser BL, Allman R. Economic evaluation of using a genetic test to direct breast cancer chemoprevention in white women with a previous breast biopsy. Appl Health Econ Health Policy. 2014;12(2):203-17.
41. Hoyle M, Green C, Thompson-Coon J, Liu Z, Welch K, Moxham T, et al. Cost-effectiveness of temsirolimus for first line treatment of advanced renal cell carcinoma. Value Health. 2010;13(1):61-8.
42. Kaambwa B, Bryan S, Jowett S, Mant J, Bray EP, Hobbs FD, et al. Telemonitoring and self-management in the control of hypertension (TASMINH2): a cost-effectiveness analysis. Eur J Prev Cardiol. 2014;21(12):1517-30.
43. Kohn CG, Parker MW, Limone BL, Coleman CI. Cost-effectiveness of ranolazine added to standard-of-care treatment in patients with chronic stable angina pectoris. Am J Cardiol. 2014;113(8):1306-11.
44. Kondo M, Yamagata K, Hoshi SL, Saito C, Asahi K, Moriyama T, et al. Cost-effectiveness of chronic kidney disease mass screening test in Japan. Clin Exp Nephrol. 2012;16(2):279-91.
45. Kongnakorn T, Lanitis T, Annemans L, Thijs V, Goethals M, Marbaix S, et al. Stroke and systemic embolism prevention in patients with atrial fibrillation in Belgium: comparative cost effectiveness of new oral anticoagulants and warfarin. Clin Drug Investig. 2015;35(2):109-19.
46. Kreidieh B, Manero MR, Cortez SH, Schurmann P, Valderrabano M. The Cost Effectiveness of LAA Exclusion. J Atr Fibrillation. 2016;8(5):1374.
47. Krzyzanowska MK, Earle CC, Kuntz KM, Weeks JC. Using economic analysis to evaluate the potential of multimodality therapy for elderly patients with locally advanced pancreatic cancer. Int J Radiat Oncol Biol Phys. 2007;67(1):211-8.
48. Kwon JS, Carey MS, Goldie SJ, Kim JJ. Cost-effectiveness analysis of treatment strategies for Stage I and II endometrial cancer. J Obstet Gynaecol Can. 2007;29(2):131-9.
49. Lairson DR, Parikh RC, Cormier JN, Chan W, Du XL. Cost-utility analysis of chemotherapy regimens in elderly patients with stage III colon cancer. Pharmacoeconomics. 2014;32(10):1005-13.
50. Lee CI, Cevik M, Alagoz O, Sprague BL, Tosteson AN, Miglioretti DL, et al. Comparative effectiveness of combined digital mammography and tomosynthesis screening for women with dense breasts. Radiology. 2015;274(3):772-80.
51. Masterton RG, Casamayor M, Musingarimi P, van Engen A, Zinck R, Odufowora-Sita O, et al. De-escalation from micafungin is a cost-effective alternative to traditional escalation from fluconazole in the treatment of patients with systemic Candida infections. J Med Econ. 2013;16(11):1344-56.

© 2024 Powell-Wiley TM et al. *JAMA Health Forum.*

52. Melnikow J, Birch S, Slee C, McCarthy TJ, Helms LJ, Kuppermann M. Tamoxifen for breast cancer risk reduction: impact of alternative approaches to quality-of-life adjustment on cost-effectiveness analysis. Med Care. 2008;46(9):946-53.
53. Morais J, Aguiar C, McLeod E, Chatzitheofilou I, Fonseca Santos I, Pereira S. Cost-effectiveness of rivaroxaban for stroke prevention in atrial fibrillation in the Portuguese setting. Rev Port Cardiol. 2014;33(9):535-44.
54. Murphy JD, Chang DT, Abelson J, Daly ME, Yeung HN, Nelson LM, et al. Cost-effectiveness of modern radiotherapy techniques in locally advanced pancreatic cancer. Cancer. 2012;118(4):1119-29.
55. Pataky R, Ismail Z, Coldman AJ, Elwood M, Gelmon K, Hedden L, et al. Cost-effectiveness of annual versus biennial screening mammography for women with high mammographic breast density. J Med Screen. 2014;21(4):180-8.
56. Paz-Ares L, del Muro JG, Grande E, Diaz S. A cost-effectiveness analysis of sunitinib in patients with metastatic renal cell carcinoma intolerant to or experiencing disease progression on immunotherapy: perspective of the Spanish National Health System. J Clin Pharm Ther. 2010;35(4):429-38.
57. Pietzsch JB, Liu S, Garner AM, Kezirian EJ, Strollo PJ. Long-Term Cost-Effectiveness of Upper Airway Stimulation for the Treatment of Obstructive Sleep Apnea: A Model-Based Projection Based on the STAR Trial. Sleep. 2015;38(5):735-44.
58. Rachapelle S, Legood R, Alavi Y, Lindfield R, Sharma T, Kuper H, et al. The cost-utility of telemedicine to screen for diabetic retinopathy in India. Ophthalmology. 2013;120(3):566-73.
59. Rupnow MF, Chang AH, Shachter RD, Owens DK, Parsonnet J. Cost-effectiveness of a potential prophylactic Helicobacter pylori vaccine in the United States. J Infect Dis. 2009;200(8):1311-7.
60. Schaufler TM, Wolff M. Cost effectiveness of preventive screening programmes for type 2 diabetes mellitus in Germany. Appl Health Econ Health Policy. 2010;8(3):191-202.
61. Scotland GS, McNamee P, Fleming AD, Goatman KA, Philip S, Prescott GJ, et al. Costs and consequences of automated algorithms versus manual grading for the detection of referable diabetic retinopathy. Br J Ophthalmol. 2010;94(6):712-9.
62. Sharaiha RZ, Freedberg DE, Abrams JA, Wang YC. Cost-effectiveness of chemoprevention with proton pump inhibitors in Barrett's esophagus. Dig Dis Sci. 2014;59(6):1222-30.
63. Wilson FA, Villarreal R, Stimpson JP, Pagan JA. Cost-effectiveness analysis of a colonoscopy screening navigator program designed for Hispanic men. J Cancer Educ. 2015;30(2):260-7.
64. Wisloff T, Hagen G, Klemp M. Economic evaluation of warfarin, dabigatran, rivaroxaban, and apixaban for stroke prevention in atrial fibrillation. Pharmacoeconomics. 2014;32(6):601-12.
65. Liu M, Wu L, Ming Q. How Does Physical Activity Intervention Improve Self-Esteem and Self-Concept in Children and Adolescents? Evidence from a Meta-Analysis. PLOS ONE. 2015;10(8):e0134804.
66. Wu B, Kun L, Liu X, He B. Cost-effectiveness of different strategies for stroke prevention in patients with atrial fibrillation in a health resource-limited setting. Cardiovasc Drugs Ther. 2014;28(1):87-98.
67. Xie F, O'Reilly D, Ferrusi IL, Blackhouse G, Bowen JM, Tarride JE, et al. Illustrating economic evaluation of diagnostic technologies: comparing Helicobacter pylori screening strategies in prevention of gastric cancer in Canada. J Am Coll Radiol. 2009;6(5):317-23.
68. Yang KY, Caughey AB, Little SE, Cheung MK, Chen LM. A cost-effectiveness analysis of prophylactic surgery versus gynecologic surveillance for women from hereditary non-polyposis colorectal cancer (HNPCC) Families. Fam Cancer. 2011;10(3):535-43.

© 2024 Powell-Wiley TM et al. *JAMA Health Forum.*

69. Zhou HJ, Dan YY, Naidoo N, Li SC, Yeoh KG. A cost-effectiveness analysis evaluating endoscopic surveillance for gastric cancer for populations with low to intermediate risk. PLoS One. 2013;8(12):e83959.

© 2024 Powell-Wiley TM et al. *JAMA Health Forum.*
