# Supplementary material for: Health and Economic Value of Eliminating Socioeconomic Disparities in US Youth Physical Activity
Source: JAMA Health Forum. 2024 Mar 15;5(3):e240088. doi: 10.1001/jamahealthforum.2024.0088 (PMC10943408; doi:10.1001/jamahealthforum.2024.0088)
Supplement: Supplement 2. — Data Sharing Statement [file jamahealthforum-e240088-s002.pdf]

Data Sharing Statement

Powell-Wiley. Health and Economic Value of Eliminating Socioeconomic Disparities in US Youth Physical Activity. *JAMA Health Forum*. Published March 15, 2024. doi:10.1001/jamahealthforum.2024.0088

Data
Data available: No

Additional Information
Explanation for why data not available: We did not conduct primary data collection for this study.
